# MiR-361-5p suppresses chemoresistance of gastric cancer cells by targeting FOXM1 via the PI3K/Akt/mTOR pathway

**DOI:** 10.18632/oncotarget.23513

**Published:** 2017-12-20

**Authors:** Lei Tian, Zhifeng Zhao, Ling Xie, JinPeng Zhu

**Affiliations:** ^1^ Department Gastroenterol, Jinzhou Medical University, Affilliated Hospital 1, Jinzhou 121000, Liaoning Province, Peoples Republic of China; ^2^ Department Gastroenterol, Zhongguo Medical University, Affilliated Hospital 4, Shengyang 110000, Liaoning Province, Peoples Republic of China; ^3^ Department Anatomy, Jinzhou Medical University, Jinzhou 121000, Liaoning Province, Peoples Republic of China

**Keywords:** MiR-361-5p, autophagy, chemoresistance, FOXM1, PI3K/Akt/mTOR

## Abstract

Gastric cancer is a prevalent cancer and chemotherapy is a main treatment for patients. Docetaxel is commonly used as a chemotherapeutic drug for gastric cancer patients. With the increasing emergence of docetaxel resistance, exploring the mechanism of chemoresistance may improve prognosis of patients. In this study, we found that overexpressed miR-361-5p suppressed chemoresistance to docetaxel of gastric cancer cells (SGC-7901, MKN-28) by decreasing IC_50_ values of docetaxel while increasing cell apoptosis rate, especially in docetaxel resistant SGC-7901 cells. Further researches revealed that overexpressed miR-361-5p inhibited chemoresistance through inhibiting autophagy with a characteristic of declined number of LC3^+^ puncta, decreased expression of Beclin-1 and the ratio of LC3 II/I and increased expression of p62. Bioinformatics study and Luciferase reporter assay indicated that FOXM1 was a target of miR-361-5p and FOXM1 was negatively regulated by miR-361-5p in gastric cancer. Simultaneously, overexpression of FOXM1 counteracted the inhibitory effects of miR-361-5p on chemoresistance of gastric cancer cells through activating autophagy, further certifying the targeting relationship between the two. Moreover, overexpressed miR-361-5p activated the PI3K/Akt/mTOR pathway. The adding of PI3K inhibitor LY294002 played an opposite role to miR-361-5p mimic by inducing autophagy and chemoresistance to docetaxel of gastric cancer cells compared with docetaxel + miR-361-5p mimic group, indicating that miR-361-5p suppressed autophagy-induced chemoresistance via the PI3K/Akt/mTOR pathway in gastric cancer cells. In conclusion, we found that miR-361-5p suppressed autophagy-induced chemoresistance of gastric cancer cells through targeting FOXM1 via the PI3K/Akt/mTOR pathway, providing a foundation for the mechanism research and treatment of gastric cancer.

## INTRODUCTION

Gastric cancer (GC) is one of the most prevalent cancers around the world, accounting for almost 10% of all cancer death [1]. Because the clinical symptom is not obvious at early stage, patients with GC are often detected at late stage, thus causing an extremely low 5-year survival rate [2]. Surgery is considered as the main treatment for patients with GC. However, for patients who miss the best time for surgery, palliative chemotherapy is the main choice of treatment. During the past decades, several new-generation cytotoxic agents including docetaxel have been used for the treatment of GC [3]. Docetaxel has been reported to promote the assembly and stabilization of microtubules to inhibit the depolymerization in the treatment of GC [4, 5]. However, obvious resistance to docetaxel has been reported in some types of tumors including prostate cancer, breast cancer, GC and nasopharyngeal carcinoma, seriously limiting the treatment effect of docetaxel [4, 6–9]. Therefore, inhibiting chemoresistance to docetaxel in GC may be a key role for improving therapeutic effects for GC patents.

Autophagy is a cellular process which delivers intracellular aggregated or misfolded proteins to the lysosomal compartment where they are degraded and recycled [10]. Previous studies reported that activation of autophagy enhanced chemoresistance in various cancers. Fukuda's study indicated that inhibition of autophagy suppressed proliferation and overcame Cisplatin resistance of endometrial cancer cells [11]. A study by Zhang revealed that inhibition of TRPC5-induced autophagy suppressed drug resistance to Adriamycin in breast carcinoma [12]. However, detailed contributions of autophagy to chemoresistance of GC cells are still limited and require for further investigations.

MicroRNAs (miRNAs), a group of small non-coding RNAs with 20-22 nucleotides, are reported to regulate the expression of specific genes [13]. Accumulated evidence indicated that miRNAs were involved in a great number of cellular processes including proliferation, apoptosis, migration and invasion during tumor progression [14, 15]. Previous studies showed that some miRNAs such as miR-608 and miR-204 suppressed chemoresistance of tumor cells [16, 17]. MiR-361-5p, one of the miRNAs, was found to function as a tumor suppressor in various tumors. Previous study showed that overexpressed miR-361-5p inhibited tumor growth of hepatocellular carcinoma in nude mice significantly [18]. However, whether miR-361-5p can regulate chemoresistance of tumor cells is still unclear.

MiRNAs are reported to regulate the expression of their target genes to accomplish their effects. In our presents study, we found that FOXM1, a newly unified family member of Forkhead transcription factor, was one of the target genes of miR-361-5p [19]. FOXM1 was reported to confer resistance to herceptin and paclitaxel by altering microtubule dynamics to protect tumor cells from paclitaxel-induced apoptosis in breast cancer [20]. As both docetaxel and FOXM1 affected the microtubules dynamics, we suggested that FOXM1 might involve in chemoresistance to docetaxel, however, little research has been carried out.

In our present study, we explored the mechanism of miR-361-5p/FOXM1 axis in regulating the chemoresistance of GC cells. We found that miR-361-5p suppressed autophagy-induced chemoresistance of GC cells through targeting FOXM1 via the PI3K/Akt/mTOR pathway, trying to find a new direction for the treatment of GC.

## RESULTS

### MiR-361-5p suppresses chemoresistance of gastric cancer cells to docetaxel

**Figure 1:**
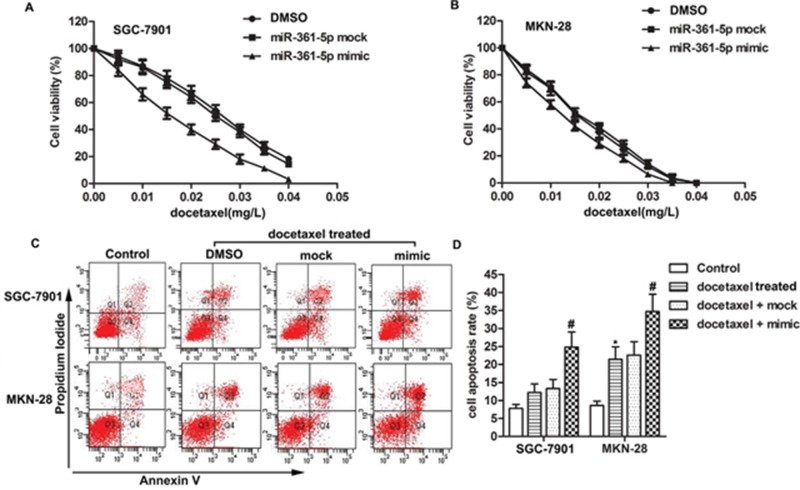
MiR-361-5p suppresses chemoresistance of gastric cancer cells to docetaxel GC cells were transfected with miR-361-5p mimic or miR-361-5p mock respectively. **(A-B)** Cell viability of transfected GC cells was tested by MTT assay. **(C-D)** Cell apoptosis was detected by flow cytometric analysis. The bars showed means ± SD of three independent experiments. P^*^ < 0.05 compared with control group, *P^#^* < 0.05 compared with docetaxel + mock group.

We initiated our study by investigating whether there was an association between miR-361-5p and chemoresistance to docetaxel of GC cells. SGC-7901 cells which were resistant to docetaxel and MKN-28 cells which were sensitive to docetaxel were treated with different concentrations of docetaxel and then cell viability was detected by MTT assay. We observed that docetaxel worked as an anti-tumor regent by suppressing cell viability significantly in a dose-dependent manner and the cell viability of SGC-7901 was higher than docetaxel sensitive cell line MKN-28 under the same concentration of docetaxel. Moreover, the half maximal inhibitory concentration (IC_50_) values of docetaxel were approximately 0.025 mg/L and 0.015 mg/L in miR-361-5p mock transfected SGC-7901 cells and miR-361-5p mimic transfected SGC-7901 cells, respectively. However, the IC_50_ in miR-361-5p mock transfected and miR-361-5p mimic transfected MKN-28 cells were approximately 0.015 mg/L and 0.013 mg/L respectively. These results suggested that overexpression of miR-361-5p increased the sensibility to docetaxel of gastric cancer cells and the promoting role was more obviously in docetaxel resistant cells (Figure 1A-1B). Apart from that, results from cell apoptosis assay indicated that overexpression of miR-361-5p increased cell apoptosis rate compared with docetaxel + mock group in both docetaxel treated SGC-7901 and MKN-28 cells (Figure 1C-1D, P^*^ < 0.05, *P^#^* < 0.05). Docetaxel resistant cells SGC-7901 were chosen for following experiments. In conclusion, our results suggested that overexpressed miR-361-5p inhibited chemoresistance of GC cells to docetaxel.

### Overexpressed miR-361-5p suppresses chemoresistance of gastric cancer cells by inhibiting autophagy

**Figure 2:**
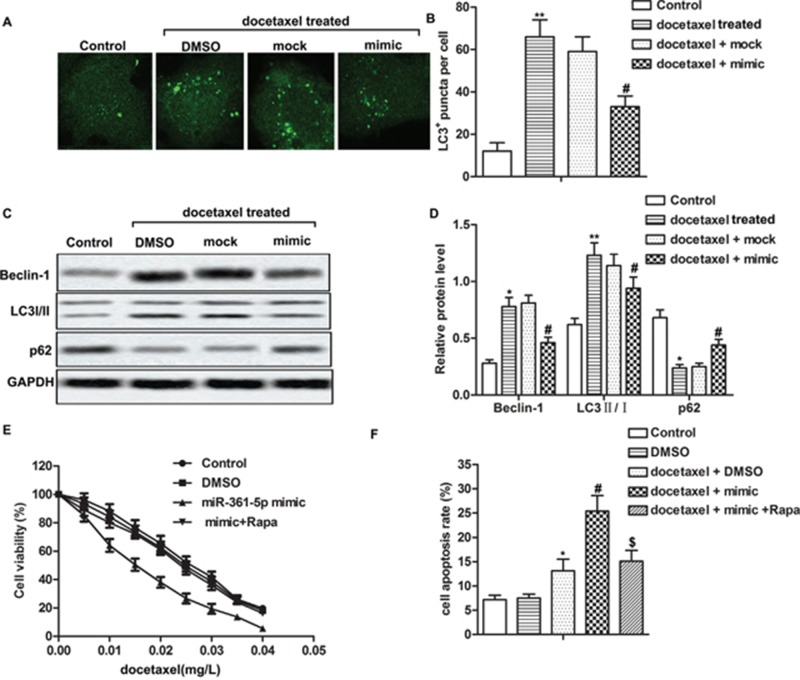
Overexpressed miR-361-5p suppresses chemoresistance of gastric cancer cells by inhibiting autophagy SGC-7901 transfected with miR-361-5p mimic or miR-361-5p mock respectively and received docetaxel treatment or DMSO treatment alone. **(A-B)** LC3^+^puncta per cell was detected through fluorescence microscopy. **(C-D)** Relative expression of Beclin-1, LC3 I/II and p62 was detected by western blot. GAPDH was used as an endogenous reference. **(E)** Cell viability was tested by MTT assay. **(F)** Cell apoptosis was detected by flow cytometric analysis. The bars showed means ± SD of three independent experiments. P^*^ < 0.05, P^**^ < 0.01 compared with control group, *P^#^* < 0.05 compared with docetaxel + mock group, *P^$^* < 0.05 compared with docetaxel + mimic group.

Accumulating evidence supported that chemoresistance of cancer cells was correlated with autophagy [21]. Therefore, we investigated whether miR-361-5p suppressed chemoresistance of GC through autophagy in our present study. The localization of LC3 to autophagosome formation was assessed through GFP-LC3 expression. Our data showed that the number of LC3^+^ puncta significantly increased after docetaxel treatment compared with control group. However, overexpressed miR-361-5p reduced the number of LC3^+^ puncta induced by docetaxel treatment compared with docetaxel + mock group markedly (Figure 2A-2B, P^**^ < 0.01, *P^#^* < 0.05). Then the expression of autophagy-related proteins was measured by western blot. Docetaxel treatment increased the expression of Beclin-1 and the ratio of LC3 II/I while decreased the expression of p62 compared with control group. Overexpressed miR-361-5p decreased the expression of Beclin-1 and the ratio of LC3 II/I while increased the expression of p62 compared withdocetaxel + mock group significantly, suggesting that miR-361-5p suppressed chemoresistance of GC cells through inhibiting autophagy (Figure 2C-2D, P^**^ < 0.01, *P^#^* < 0.05). In order to verify our conjecture, autophagy inducing reagent rapamycin (Rapa) was used in our study. Because Rapa was dissolved in DMSO, thus DMSO group was used as control and we found that DMSO didn't have significant effect on cell viability and apoptosis rate. We observed that the IC_50_ values were approximately 0.015 mg/L and 0.025 mg/L in miR-361-5p mimic group and mimic + Rapa group respectively, indicating that activation of autophagy by Rapa decreased the sensibility to docetaxel of overexpressed miR-361-5p GC cells (Figure 2E). Besides that, activation of autophagy abolished the promoting effect of miR-361-5p on cell apoptosis compared with docetaxel + mimic group remarkably (Figure 2F, P^*^ < 0.05, *P^#^* < 0.05), suggesting that activation of autophagy increased chemoresistance of GC cells to docetaxel. Taken together, these results supported that overexpressed miR-361-5p suppressed chemoresistance of GC cells through inhibiting autophagy.

### FOXM1 is a target of miR-361-5p in gastric cancer cells

**Figure 3:**
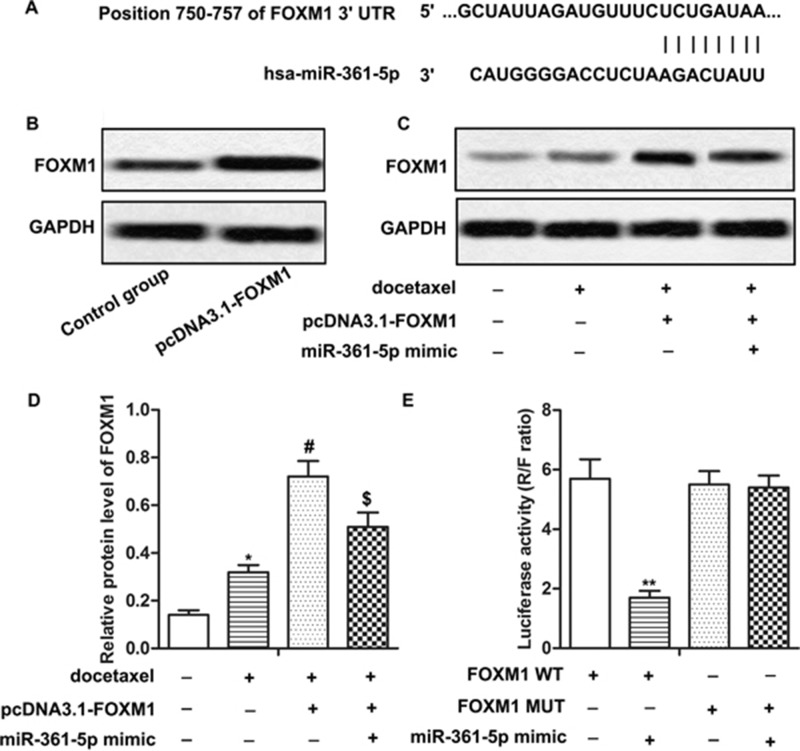
FOXM1 is a target of miR-361-5p in gastric cancer cells **(A)** Bioinformatics analysis of miR-361-5p and FOXM1. **(B)** Relative protein level of FOXM1 in pcDNA3.1-FOXM1 group and control group was detected by western blot. GAPDH was used as an endogenous reference. **(C-D)** Relative protein level of FOXM1 of transfected SGC-7901 with docetaxel treatment in different groups was detected by western blot. GAPDH was used as an endogenous reference. **(E)** Results of Luciferase reporter assay. The bars showed means ± SD of three independent experiments. P^*^ < 0.05, P^**^ < 0.01 compared with control group, *P^#^* < 0.05 compared with docetaxel treated group, *P^$^* < 0.05 compared with docetaxel + FOXM1 group.

In order to explore the molecular mechanisms of miR-361-5p in chemoresistance of GC cells, putative miR-361-5p targets were predicted through bioinformatics analysis. The predicted results showed that FOXM1 was one of the potential targets of miR-361-5p (Figure 3A). SGC-7901 cells were transfected with pcDNA3.1-FOXM1 for overexpression of FOXM1 (Figure 3B). Results from western blot showed that the expression of FOXM1 was increased by docetaxel treatment compared with control group and was decreased by the adding of miR-361-5p mimic compared with pcDNA3.1-FOXM1 group under the application of docetaxel, indicating that the expression of FOXM1 was negatively regulated by miR-361-5p (Figure 3C-3D, P^*^ < 0.05, *P^#^* < 0.05, *P^$^* < 0.05). Moreover, the luciferase reporter assay showed that co-transfection with miR-361-5p mimic and FOXM1 WT in SGC-7901 cells led to a significant decrease in luciferase activity. However, co-transfection with FOXM1 MUT and miR-361-5p mimic didn't show significant difference compared with cells transfected with FOXM1 MUT (Figure 3E, P^**^ < 0.01), confirming the targeting relationship between FOXM1 and miR-361-5p. In summary, our data elucidated that FOXM1 was a target of miR-361-5p and the expression of FOXM1 was negatively regulated by miR-361-5p in GC cells.

### Overexpression of FOXM1 decreases the inhibitory effects of miR-361-5p on autophagy-induced chemoresistance in gastric cancer cells

**Figure 4:**
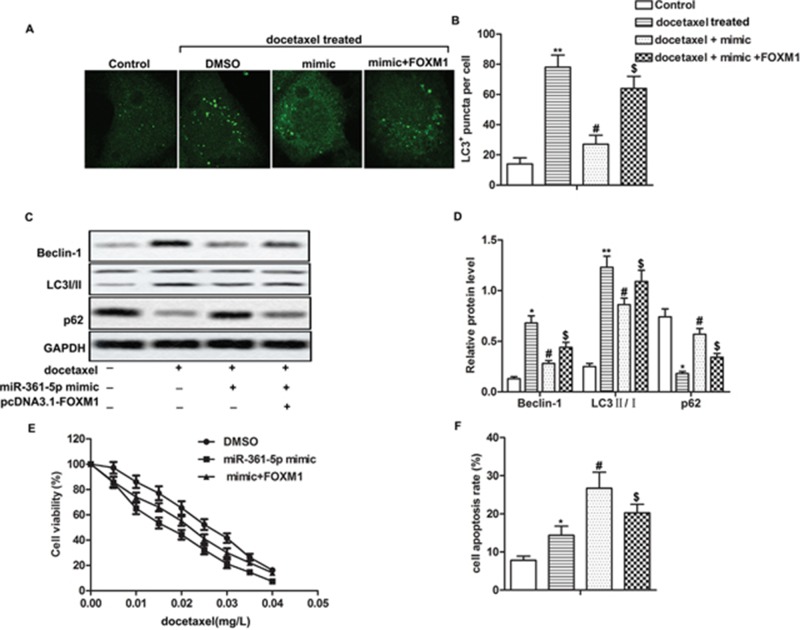
Overexpression of FOXM1 decreases the inhibitory effects of miR-361-5p on autophagy-induced chemoresistance in gastric cancer cells SGC-7901 cells transfected with miR-361-5p mimic or mimic + pcDNA3.1-FOXM1 received docetaxel treatment. **(A-B)** LC3^+^ puncta per cell was detected through fluorescence microscopy. **(C-D)** Relative expression of Beclin-1, LC3 I/II and p62 was detected by western blot. GAPDH was used as an endogenous reference. **(E)** Cell viability was tested by MTT assay. **(F)** Cell apoptosis was detected by flow cytometric analysis. The bars showed means ± SD of three independent experiments. P^*^ < 0.05, P^**^ < 0.01 compared with control group, *P^#^* < 0.05 compared with docetaxel treated group, *P^$^* < 0.05 compared with docetaxel + mimic group.

Having learned the relationship between FOXM1 and miR-361-5p, we then set to explore the mechanism of miR-361-5p in chemoresistance of GC cells. Co-transfection with miR-361-5p mimic and pcDNA3.1-FOXM1 increased the number of LC3^+^ puncta, the expression of Beclin-1 and the ratio of LC3 II/I while decreased the expression of p62 compared with docetaxel + mimic group, indicating that overexpressed FOXM1 weaken the inhibitory effect of miR-361-5p on autophagy (Figure 4A-4D, P^*^ < 0.05, P^**^ < 0.01, *P^#^* < 0.05, *P^$^* < 0.05). Besides that, our data showed that IC_50_ values were approximately 0.015 mg/L and 0.025 mg/L in miR-361-5p mimic group and mimic + FOXM1 group respectively and overexpressed FOXM1 inhibited cell apoptosis compared with docetaxel + mimic group remarkably, suggesting that overexpressed FOXM1 counteracted the inhibitory effect of miR-361-5p on chemoresistance of GC cells (Figure 4E-4F, P^*^ < 0.05, *P^#^* < 0.05, *P^$^* < 0.05). Taken together, these data clearly indicated that overexpression of FOXM1 counteracted the inhibitory effects of miR-361-5p on autophagy-induced chemoresistance in GC cells.

### MiR-361-5p suppresses autophagy-induced chemoresistance via the PI3K/Akt/mTOR pathway

**Figure 5:**
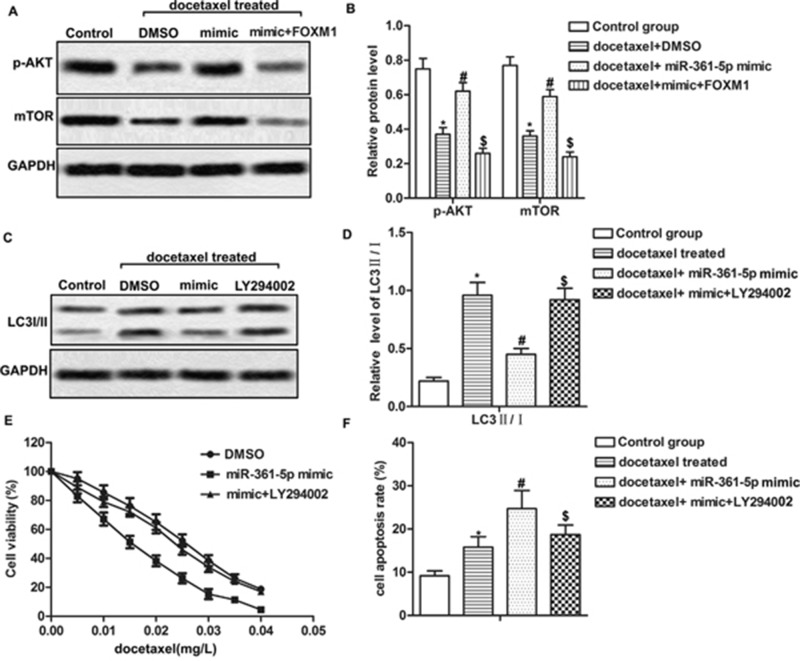
MiR-361-5p suppresses autophagy-induced chemoresistance via the PI3K/Akt/mTOR pathway MiR-361-5p mimic transfected SGC-7901 cells received docetaxel treatment with or without LY294002 treatment. **(A-B)** Relative expression of p-AKT and mTOR was detected by western blot. GAPDH was used as an endogenous reference. **(C-D)** The ratio of LC3 II/I was detected by western blot. GAPDH was used as an endogenous reference. **(E)** Cell viability was tested by MTT assay. **(F)** Cell apoptosis was detected by flow cytometric analysis. The bars showed means ± SD of three independent experiments. P^*^ < 0.05 compared with control group, *P^#^* < 0.05 compared with docetaxel treated group, *P^$^* < 0.05 compared with docetaxel + miR-361-5p mimic group.

Previous study reported that PI3K/Akt/mTOR pathway played an important role in regulating autophagy [22]. Therefore, we further explored whether miR-361-5p suppressed chemoresistance of GC cells through PI3K/Akt/mTOR pathway. The data revealed that docetaxel treatment decreased the expression of p-AKT and mTOR compared with control group. However, overexpression of miR-361-5p increased the low expression of p-AKT and mTOR caused by docetaxel while overexpressed FOXM1 decreased the expression of p-AKT and mTOR significantly compared with docetaxel + miR-361-5p mimic group, suggesting that overexpression of miR-361-5p activated while overexpressed FOXM1 suppressed PI3K/Akt/mTOR pathway in docetaxel treated GC cells (Figure 5A-5B, P^*^ < 0.05, *P^#^* < 0.05, *P^$^* < 0.05). Moreover, treatment with PI3K inhibitor LY294002 increased the ratio of LC3 II/I compared with docetaxel + miR-361-5p mimic group (Figure 5C-5D, P^*^ < 0.05, *P^#^* < 0.05, *P^$^* < 0.05). The IC_50_ values were about 0.015 mg/L and 0.025 mg/L in miR-361-5p mimic group and mimic + LY29400 group respectively, indicating that the inactivation of PI3K/Akt/mTOR pathway decreased sensibility to docetaxel in GC cells transfected with miR-361-5p mimic (Figure 5E). LY294002 treatment also decreased cell apoptosis rate compared with docetaxel + miR-361-5p mimic group (Figure 5F, P^*^ < 0.05, *P^#^* < 0.05, *P^$^* < 0.05). In conclusion, our results indicated that miR-361-5p suppressed autophagy-induced chemoresistance via the PI3K/Akt/mTOR pathway.

## DISCUSSION

In gastric cancer treatment, surgery combined with chemotherapy is one of the most common methods to improve prognosis of patients. Docetaxel, one of the chemotherapy agents, is commonly used as a single agent or in combination with other agents such as latinum and fluoropyrimidine (DCF regimen) for the treatment of cancer patients [23, 24]. Docetaxel is an active agent in advanced GC and it has also been shown to lack cross-resistance with other drugs in GC [5]. However, the resistance to docetaxel did occur and more investigations on the mechanism of chemoresistance to docetaxel of tumor cells are urgently required. Our study presented the role of miR-361-5p/FOXM1 axis in the chemoresistance to docetaxel of GC cells and tried to find a better therapeutic strategy for overcoming the resistance to docetaxel in GC.

MiRNAs have gained increasing attentions as they appeared to be involved in tumor progression. Increasing evidence supported that miRNAs acted as tumor suppressors or oncogenes and involved in post-translational regulation of gene expression [25]. Previous studies reported that a great number of miRNAs were related to chemoresistance in various cancers. For example, Rajabpour's study revealed that miR-608 suppressed gemcitabine chemoresistance through regulating the resistance genes in pancreatic cancer cells [16]. Besides that, microRNA-204 was reported to increase chemosensitivity of prostate cancer cells [17]. Recently, accumulating evidence supported that miR-361-5p acted as a tumor suppressor in many kinds of tumors. Zhuang's research indicated that down-regulation of miR-361-5p associated with aggressive clinicopathological features and unfavorable prognosis in non-small cell lung cancer [26]. It was also reported that miR-361-5p inhibited epithelial-to-mesenchymal transition through targeting Twist1 in glioma cells [27]. However, whether miR-361-5p could regulate chemoresistance of tumor cells still remained unknown. In our present study, SGC-7901 cells which were resistant to docetaxel and MKN-28 cells which were sensitive to docetaxel were chosen for our experiments. Results indicated that overexpressed miR-361-5p in SGC-7901 and MKN-28 cells decreased the IC_50_ value of docetaxel and increased cell apoptosis rate, suggesting that miR-361-5p inhibited chemoresistance to docetaxel of SGC-7901 and MKN-28 cells and the inhibiting effect was more obviously found in SGC-7901 cells. Our results elucidated that the inhibitive effect of miR-361-5p on chemoresistance in GC cell lines.

After learning the effect of miR-361-5p on chemoresistance of GC cells, we then set to explore its potential mechanism. Autophagy was a programmed cell survival mechanism which was reported to enhance chemoresistance of tumor cells. Hu's data showed that p53 was important for the activation of autophagy and suppression of p53 potentiated chemosensitivity in nutrient-deprived cholangiocarcinoma cells [28]. Activation of autophagy by Fusobacterium nucleatum promoted chemoresistance of colorectal cancer cells [29]. Similarly, our results showed that overexpressed miR-361-5p decreased the number of LC3^+^ puncta which was widely used as a marker for autophagosomes. Moreover, the expression of Beclin-1 which was necessary for the formation of autophagosomes and the ratio of LC3 II/I were decreased while the expression of p62 which cleared the autophagosome was increased by overexpressed miR-361-5p, indicating that miR-361-5p inhibited autophagy in docetaxel treated SGC-7901 cells. Besides that, activation of autophagy by Rapa counteracted the inhibitory effect of miR-361-5p on chemoresistance to docetaxel through decreasing docetaxel sensibility and cell apoptosis rate in GC cells. Our data revealed that miR-361-5p suppressed chemoresistance through inhibiting autophagy in GC cells.

Furthermore, our bioinformatics study revealed that FOXM1 was one of the potential targets of miR-361-5p. FOXM1, a proliferation specific oncogenic transcription factor, was reported to regulate microtubule dynamics to mediate chemoresistance of tumor cells [20]. Previous research discovered that inhibition of FOXM1 could reverse docetaxel resistance in GC [4]. FOXM1 also mediated resistance to herceptin and paclitaxel in breast cancer [20]. Another study from Hou elucidated that overexpression of miR-361-5p suppressed lung cancer proliferation and invasion by targeting FOXM1 [30]. Consistent with previous researches, we found that the expression of FOXM1 was negatively regulated by miR-361-5p. Overexpressed FOXM1 enhanced autophagy and chemoresistance to docetaxel of GC cells, suggesting that overexpression of FOXM1 weakened the inhibitory effects of miR-361-5p on autophagy-induced chemoresistance in gastric cancer cells.

PI3K/Akt/mTOR pathway was a well-known pathway involved in the regulation of autophagy. Inhibition of PI3K/Akt/mTOR pathway was reported to enhance autophagy [31]. Previous study showed that autophagy was negatively regulated by the activation of mTOR in APP/PS1 double transgenic mice [32]. In accordance with these studies, overexpressed miR-361-5p was found to activate PI3K/Akt/mTOR pathway while overexpressed FOXM1 suppressed this pathway. Apart from that, PI3K inhibitor LY294002 abolished the effect of miR-361-5p on autophagy and chemoresistance to docetaxel of GC cells compared with docetaxel + miR-361-5p mimic group, indicating that miR-361-5p suppressed autophagy-induced chemoresistance by targeting FOXM1 via the PI3K/Akt/mTOR pathway in GC cells.

Taken together, our present study demonstrated that miR-361-5p/FOXM1 axis played an important role in regulating chemoresistance to docetaxel of GC cells. MiR-361-5p suppressed autophagy-induced chemoresistance of GC cells by targeting FOXM1 via the PI3K/Akt/mTOR pathway, providing a foundation for the mechanism research and treatment of GC.

## MATERIALS AND METHODS

### Cell lines and culture conditions

GC cell lines (SGC-7901, MKN-28) were purchased from American Type Culture Collection (ATCC). All the cells were cultured in DMEM (Gibco, Rockville, MD) medium supplemented with 10% FBS at 37°C.

Docetaxel (Selleckchem, Houston, TX, USA) was dissolved in DMSO and diluted to a final concentration of 0.01, 0.02, 0.03 and 0.04 mg/L. Rapamycin (Sigma, St. Louis, MO, USA), an autophagy activator, was used to treat cells for 24 h at the concentration of 100 nM. LY294002 (Selleck Inc, USA), a PI3K inhibitor, was used to treat cells for 24 h at the concentration of 10 μM.

### Transfection

For miR-361-5p transfection, cells were seeded into 96-well plate to reach 60% confluence state. MiR-361-5p mimic or miR-361-5p mock (GenePharma, Shanghai, China) was transfected into SGC7901 cells using Lipofectamine 2000 reagent (Life Technologies Corporation, Carlsbad, CA, USA) according to manufacturer's protocol.

The human FOXM1 expression vector pcDNA3.1-FOXM1 was transfected into cells using Lipofectamine 2000 reagent in accordance with the manufacturer's protocol.

### MTT assay

For MTT assays, 5×10^3^ cells were seeded into 96-well plates in triplicate and cultured with 100 μL of fresh medium which contained different concentrations of docetaxel for 48 h. Then, MTT solution (20 μL, 5 mg/ml in PBS) was added to each wells and incubated at 37°C for 4 h. After that, the solution was removed and 200 μL of DMSO was added to each well. The optical density at 490 nm was measured by a microplate reader (Bio-Rad, Hercules, CA, USA) after 10 min of vibration mixing.

### Cell apoptosis assay

For cell apoptosis analysis, Annexin V-FITC/propidium iodide (PI) apoptosis detection kit (Multisciences, Shanghai, China) was used in our present study. Firstly, different groups of cells (3×10^5^) were collected and washed in ice-cold PBS. Then cells were resuspended and incubated with 5 μL of Annexin V-FIFC and 10 μL of PI. Cell apoptosis was analyzed in a flow cytometer (BD Biosciences).

### Fluorescence microscopy

MiR-361-5p mimic or mock transfected SGC-7901 cells were transfected with GFP-LC3 plasmid by Lipofectamine 3000™ (Invitrogen). After untreated SGC-7901 cells or transfected cells treated with or without docetaxel, the number of puncta formation of GFP-LC3 was determined under fluorescent microscopy as described before [33]. Cells with more than 5 puncta counted were considered to have accumulated autophagosomes.

### Western blot

Proteins were extracted from cells using RIPA lysis buffer (Beyotime Institute of Biotechnology, Shanghai, China) according to the manufacturer's protocol. Total proteins were separated by SDS-PAGE gel and then transferred into PVDF membranes (Millipore). After incubating with 5% skim milk for 1 h at room temperature, the membranes were incubated with primary antibodies overnight at 4°C. The primary antibodies (Cell Signaling Technology, Beverly, MA., USA) used in this study include anti-Beclin-1 (No. 4122, 1:1000), anti-LC3 I/II (No.12741, 1:1000), anti-p62 (No.88588, 1:1000), anti-FOXM1 (No.5436, 1:1000), anti-p-AKT (No.4060, 1:2000), anti-mTOR (No.2983, 1:1000) and anti-GAPDH (No.5174, 1:2500). Then, the membranes were incubated with corresponding secondary antibodies (Cell Signaling Technology, No.7076 and NO.7074, 1:4000) at room temperature for 1 h. ECL system (Bio-Rad Laboratories) was used for detection of antibody-bound proteins according to manufacturer's instructions.

### Bioinformatics method

In order to evaluate miR-361-5p's potential target genes, the following online miRNA target prediction algorithms were used: PicTar (http://www.pictar.org/), miRanda (http://www.microrna.org/microrna/home.do), TargetScan (http://www.targetscan.org/vert_71/) and Microcosm Targets (http://www.ebi.ac.uk/enright-srv/microcosm/htdocs/targets/v5/).

### Luciferase reporter assay

The fragment of FOXM1 containing the target sequence of miR-361-5p was inserted into a pmirGlO Dual-luciferase miRNA Target Expression Vector (Promega, Madison, WI, USA) to form the reporter vector FOXM1-wild-type (FOXM1-WT) while FOXM1-mutated-type (FOXM1-MUT) contained mutated binding site. Cells at 5×10^4^ were seeded into 96-well plate to reached 60% confluence. Then cells were co-transfected with FOXM1-WT or FOXM1-MUT and miR-361-5p mimics using Lipofectamine 2000. The Dual-Luciferase Reporter Assay System (Promega, Madison, WI, USA) was used for testing the luciferase activity.

### Statistical analysis

All results were presented as mean ± standard deviation (SD). Student's t test was performed to analyze the difference between groups. Statistical analysis was performed with SPSS software (version 19). Values of *P* < 0.05 were considered statistically significant.

## Abbreviations

GC: gastric cancer
miRNAs: MicroRNAs
SD: standard deviation
Rapa: rapamycin.
